# The Influence of Presentation Frames of Visualization Information for Safety on Situational Awareness under a Three-Level User-Interface Design

**DOI:** 10.3390/ijerph20043325

**Published:** 2023-02-14

**Authors:** Xiaofang Yuan, Jing Yan, Linhui Sun, Fangming Cheng, Zigu Guo, Hongzhi Yu

**Affiliations:** 1 School of Management, Xi’an University of Science and Technology, Xi’an 710054, China; 2 Research Center for Human Factors and Management Ergonomics, Xi’an University of Science and Technology, Xi’an 710054, China; 3 School of Safety Science and Engineering, Xi’an University of Science and Technology, Xi’an 710054, China

**Keywords:** framing effect, situation awareness (SA), visualization information for safety (VIS), situation-present-assessment method (SPAM), situation-awareness-rating technique (SART), eye-movement

## Abstract

To explore the influence of the construction and presentation frames of visualization information for safety (VIS) on people’s situation awareness (SA), we designed a three-level user interface (UI) of VIS based on the three-stage SA theory, including perception (SA1), comprehension (SA2), and projection (SA3). Then, 166 subjects were recruited and divided into three groups to participate in the experiment, in which SA was measured by the situation-present-assessment method (SPAM) and situation-awareness-rating technique (SART), and eye-movement data were recorded. The results show that the level−3 UI design could effectively improve the subjects’ SA levels. Although the increase in VIS displayed caused by the higher UI level led to a decrease in the perception-stage score of SA, the level−3 UI fully considered the three stages of human information processing, and helped improve the SA of the subjects; the overall SA score measured using the SART method was not significant, but the result was consistent with the SPAM. There was a framing effect on the presentation of VIS, and subjects perceived different degrees of risk under different presentation frames; that is, less risk under the positive frame, more risk under the negative frame, and a higher level of SA under the positive frame compared with the negative frame. To some extent, the nearest-neighbor-index (NNI) algorithm could be utilized to quantify subjects’ eye-tracking fixation mode. While the frames were guided by the high-level interface and the positive presentation frame, the distribution of the subjects’ gaze points was more discrete; they could grasp the relevant information more comprehensively and had a relatively high level of SA. To some extent, this study can provide a reference for the design and optimization of the VIS presentation interface.

## 1. Introduction

With the development of the Internet, big data, cloud computing, and other technologies and urbanization, information visualization has played an increasingly crucial role in the field of safety and emergency management. Visualization information for safety (VIS) is the transformation of information and knowledge related to safety risks into a visual representation, using the human ability to quickly identify views to improve cognitive efficiency and make high-quality decisions [1]. In recent years, the application of various safety-risk-monitoring technologies has gradually become popular. When VIS is presented on the platform interface, people can perceive, understand, and predict the safety-risk status of the target object promptly. For example, during the outbreak of COVID-19, a large amount of VIS emerged to help the public quickly understand the real-time development of the epidemic and predict the future evolutionary trend by promptly conveying the quantity of information and spatial distribution of the outbreak to the public [2]. However, with the increasing complexity and diversification of VIS, VIS has brought abundant information and increased people’s cognitive load. Thus, minimizing the cognitive load of the interface design is one aspect of improving its usability [3,4,5]. Weiser argued that information technology is often the enemy of calm, with countless pieces of information trying to attract attention, and, owing to the limitation of an individual’s attention resources, the usability of interface design can be achieved by minimizing user attention, improving the user’s situation awareness (SA), and minimizing their input [6]. Blackwell’s proposed framework of cognitive dimensions of notations (CDs) predicts the usability of interface design in terms of the structural properties of a notation, its properties, and the resources of an environment, providing an account of information construction that respects the value of user activity [7,8]. A good visualization of safety information can help people better obtain, store, and process information so that they can make effective decisions. Within the general context of visualization for safety information, as the actual safety status of the target object is invisible, the relevant context needs to be constructed and maintained in the brain, and the way information is constructed and represented affects people’s construction and maintenance of SA [9,10,11,12].

SA is the perception of the elements in the environment, the comprehension of their meaning, and the projection of their status in the near future within a given period of time and area of space [13]. It can be explained through the classical three-stage theory of information processing [13]. The first stage is perception (SA1); attention is the key link in this stage, and one makes a simple perception of task-relevant elements and their current state in the surrounding environment. The second stage is comprehension (SA2), where one understands how they affect the goal by integrating the elements of stage one. The third stage is projection (SA3), where one integrates the information from the first two stages to predict the future behavior and state of these elements within the context. The concept of SA first originated in the 1990s with the study of fighter pilots [14], and has since been extended to other fields [15]. It is frequently used to evaluate human–machine-interface design [16,17]. Jones and Endsley reported that 76.3% of errors were related to the perception of information, 20.3% of errors were related to the comprehension of information, and 3.4% of errors were related to “failure to project the situation into the future” [18]. Researchers have found that presenting more information can make participants feel overconfident and lead to less accurate decisions [19]. However, some studies have concluded that people’s decisions depend on limited information, and that “less is more” [20]. Thus, it can be concluded that the construction of information has an impact on SA. As the VIS elements presented become more complex, it is worth investigating how to construct them to better accommodate people’s cognitive processing.

In addition, how information is presented via the interface largely influences SA by determining how much information can be acquired, how accurately it can be acquired, and to what degree it is compatible with people’s SA needs [13]. Recently, Rakhra [21] and Fu [22] found that different displaying formats for the same visual elements could have an impact on people’s SA and cognitive efficiency. Some scholars have also studied the effects of information presentation on individual cognition and decision making from the perspective of information-representation frames, typically through the framing effect [23,24,25,26]. The framing effect refers to the phenomenon that equivalent descriptions of the same issue lead to different decision preferences. The traditional verbal framing effect was first demonstrated by Tversky and Kahneman, based on the Asian disease problem [23]. Subsequent scholars have explored its prevalence and influencing factors [24,25]. Among the types of framing effects, the attribute-framing effect [26] occurs when a single attribute of an object or event is carried over in the positive or negative description, resulting in a different perception of quality for consumers. For example, research has shown that when beef is described as “75% lean” and “25% fat,” the result is a different perception of quality for consumers, with more people preferring “75% lean” in the positive frame [26]. However, few scholars have studied the framing effect of visualization-information representation and explored it in conjunction with the concept of SA. In conjunction with the objective of this paper, frames can also be understood as different presentations of the same visualization information. When VIS elements are represented in different frames, it is worth investigating whether people perceive risks differently at the level of perception, comprehension, and projection of the information.

Thus, we designed a three-level interface for VIS presentation based on the three-stage SA theory, and, based on this, we manipulated the representation frames of VIS and adopted the objective, subjective, and process-integrated measurement method to investigate whether and how the construction and representation of VIS under the three-level UI design influence people’s SA. We also aimed to provide a theoretical basis for the design and optimization of VIS presentation interfaces, to some extent. The following hypotheses are proposed: (1) The construction of VIS elements in the interface has an effect on an individual’s SA, and different UI levels have different effects on the three stages of information processing, that is, perception, comprehension, and prediction, which in turn affect an individual’s overall SA. (2) There is a framing effect in the presentation of VIS, and different presentation frames affect individual’s cognitive processing of the three stages of perception, comprehension, and prediction of information, which in turn affects their SA.

## 2. Design of VIS Elements and the Three-Level UI

### 2.1. Design of VIS Elements

**Figure 1:**
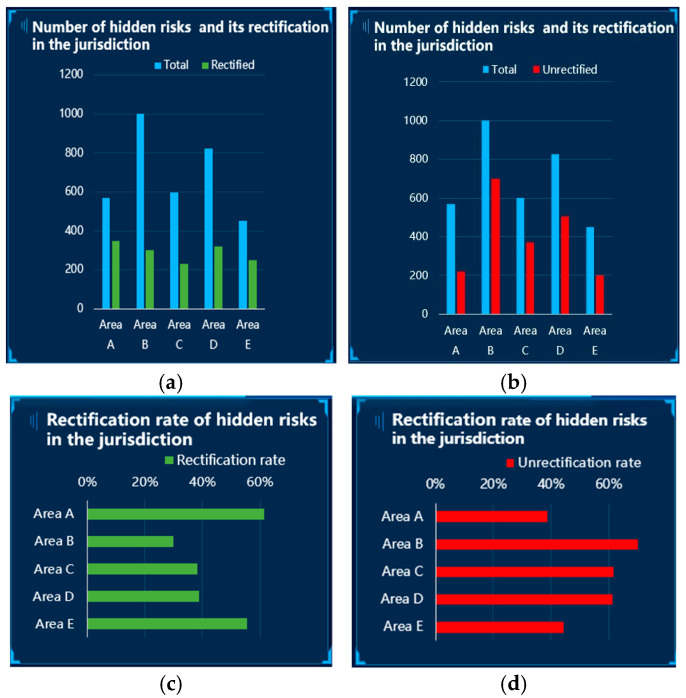
Main VIS elements after manipulating the attribute frames: (**a**,**c**) are positive frames; (**b**,**d**) are negative frames.

**Figure 2:**
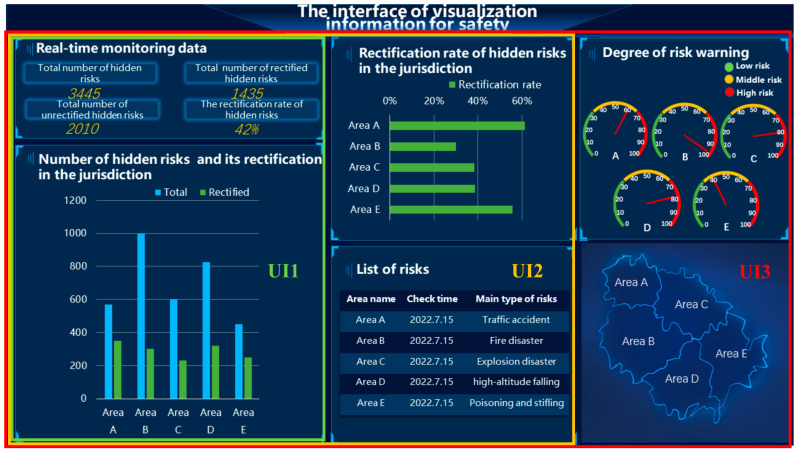
Three-level UI design for VIS presentation (English version). Note. This figure shows the UIs employed in the study. The green-line box is the level-1 UI, the orange-line box is the level-2 UI, and the red-line box is the level-3 UI.

By investigating the existing VIS presentation interface, we found that the common VIS included information such as the basic information of hidden risks, the number of hidden risks and their rectification, and the degree of risk warning. Combined with the three-stage SA theory, the main VIS elements used in this study were selected, including the statistical chart for the number of hidden risks and their rectification, the statistical chart of the hazard-rectification rate, and the risk-warning chart in the jurisdiction, which are presented in this paper in a vertical bar chart, horizontal bar chart, and dashboard chart, respectively (Figure 1 and Figure 2). Combined with the theory of framing effect, the attribute frames were manipulated using the “number of rectified hazards” and “number of unrectified hazards,” “rectification rate of hazards,” and “unrectified rate of hazards” (Figure 1). To reproduce the VIS-presentation-interface reality, we show the corresponding bar charts in green and red colors to represent “safe” and “danger,” respectively. Previous studies revealed that red was recognized globally as the color for danger, and this was consistent across cultures and occupations; green, although not recognized globally as the color for “safe,” represented “low risk” in most cultures and “safe” in the Chinese cultural context [27]. Therefore, in this study, green and red colors were chosen to represent the “safe” and “danger” messages, respectively.

### 2.2. Three-Level UI Design for VIS Presentation

Based on the three-stage-SA theory and the actual situation, a three-level UI for the VIS presentation was designed (see Figure 2).

Level-1 UI (UI1): this level of interface presents a small number of VISs. At this level, one gets an intuitive sense of the total number of hazards currently in the jurisdiction and the number of hazards that have been rectified.

Level-2 UI (UI2): based on UI1, certain ratio information is added to help people understand the status of the rectification of hidden hazards in each jurisdiction.

Level-3 UI (UI3): based on UI1 and UI2, certain probabilistic risk information is added to help people better predict the future state of risks in each jurisdiction.

## 3. Methods

### 3.1. The Measurement of SA

#### 3.1.1. Three-Level UI Design for VIS Presentation

Five SA-measurement methods are commonly used: subjective measures (SART), objective measures (SAGAT and SPAM), process measures (such as eye-tracking), and performance measures [28]. In this study, the situation-present-assessment method (SPAM), situation-awareness-rating technique (SART), and eye-tracking technique were used to measure the subjects’ level of SA in each presentation frame, based on the three-stage SA theory. Among them, SPAM is a real-time probe technique that measures participants’ SA using online-probe questions related to the task without freezing the scenario, which records response accuracy and latency to measure SA [29]. As SPAM focuses on the ability to locate information in the situation and does not require freezing the task performed by the subject, compared with the situation-awareness-global-assessment technique (SAGAT), which reduces the intrusiveness of the task [30], it was chosen as the primary SA-measurement method because of the purpose of this study and the complexity of VIS presented. SART [31], a subjective measurement method of SA conducted after the experiment, is a multidimensional scale, and the most widely used is the 3D-SART, which mainly includes three dimensions: demand for attentional resources (AD), supply of attentional resources (AS), and understanding of the current situation (SU). To make the research results more reliable, we further adopted the subjective SART to assess the subjects’ SA. Furthermore, visualization information is a form of visual–spatial information, and eye tracking is an effective method for studying a person’s cognition, because it captures the vision of the individual. Eye-movement patterns reflect different information-processing processes, among which SA-process measures have been validated in pilot studies [32,33,34]. Moreover, for the framing effect, different representations may activate different adaptors, and accordingly, different factors, such as context, may activate different domain-specific adaptors [35]. Accordingly, this study also combined eye-tracking techniques to measure people’s visual processing of interface information and investigate how the presentation frame of VIS affects their attention allocation under the three-level UI design.

#### 3.1.2. Measurement Material of SA

(1) Design the Probe Questions of SPAM

The SPAM questionnaire was designed based on the study by Jones et al. [36] and the context of this study, with a total of 10 items. Of these, items Q1–Q6 and Q8–Q10 were measured by accuracy, which was obtained based on the VIS presented in the interface, while Q7 was measured by a 7-point Likert scale, and Q10 was a ranking of the likelihood of an accident occurring in the area (e.g., BCDAE). That is, area B is the most likely to see an accident, followed by areas C, D, A, and E (Table 1).

(2) Design the Subjective Scales of SART

Three-dimensional SART scales were designed based on the studies of Taylor [31] and Su et al. [37], containing a total of 10 questions, all measured using the 7-point Likert scale (Table 2).

### 3.2. Experimental Design

#### 3.2.1. Independent Variables

In this study, a two-factor mixed experiment was adopted, with factor 1 being the UI level (between-subject variable), containing three levels: UI1, UI2, and UI3. Factor 2 is the presentation frame of VIS (within-subject variable) and consists of four levels: the positive–positive frame (PP), the positive–negative frame (PN), the negative–positive frame (NP), and the negative–negative frame (NN).

By manipulating the presentation frame of VIS at each UI level, we incorporated a total of 10 relevant contexts into the experiment. In UI1, the presentation frame of the total number of rectified hazards in the jurisdiction was manipulated, and only two relevant contexts existed at this level, namely PP and NN. The influence of the framing effect is mainly focused on the perception and comprehension stages, which can be illustrated by the dual-system theory; that is, there are two systems of intuitive and inferential judgments involved in the process of people making judgments about problems, where system 1 adopts a heuristic processing mode, similar to perception, while system 2 requires more computation, and adopts an analytical processing mode [38,39]. Thus, further, manipulating the presentation frame for the rectification rate of potential hazards in the UI2, and with the combination of UI1, four relevant contexts exist at this level, namely PP, PN, NP, and NN. UI3 adds the prediction module to UI2, with four relevant contexts, namely PP, PN, NP, and NN.

#### 3.2.2. Dependent Variable

The dependent variable in this study is the SA of subjects toward different situations, which was comprehensively measured by objective, subjective, and process methods, as follows.

(1) The three-stage and total scores obtained by SPAM. Based on the E-prime experimental platform, the proportion of correct responses to the probe questions was recorded to measure the SPAM score of SA, that is, the total SPAM score of SA = the number of correct responses/total number of probe questions × 100, and the stage scores (SSA1, SSA2, SSA3) were also calculated.
(2) The three-dimensional and total scores obtained by SART (post-test score of SA). The subjective SART score was calculated based on the three-dimensional scores, including the score for attention demand (SAD), the score for attention supply (SAS), and the score for situational understanding (SSU), that is, SSA = SSU − (SAD − SAS).
(3) Eye-movement indexes: the nearest neighbor index (NNI), the average regression count in the area of interest (RCAOI), the average fixation count in the area of interest (FCAOI), and the average fixation duration in the area of interest (FDAOI) were selected as eye-movement indexes. Among them, the NNI-fixation index is a commonly used clustering algorithm, based on the distance between gaze points within a region, which is influenced by the subjects’ SA level and visual-search strategy, measuring the spatial dispersion of their gaze points [40]. When NNI < 1, the points are aggregated; when NNI = 1, the points are randomly dispersed; when NNI > 1, the points are regularly dispersed. The gaze and look-back behavior for a visual element reflect the attention allocation, acquisition, and storage [34,41]. The regression count reflects the individual’s level of reprocessing of information [42], while the fixation count reflects the subject’s ability to extract information during the task; the higher the number of gaze points, the more attention the subject pays to the area and the more useful information the subject can extract [43]. The average fixation duration is generally used for coding tasks. The longer the fixation duration, the more difficult it is to extract information, the greater the cognitive load, or the more appealing the target [44]. Thus, the abovementioned eye-movement indicators were chosen to measure the overall and local distribution of attention and its correlation with the level of SA in each UI level and presentation frame.

#### 3.2.3. Control Variables

**Figure 3:**
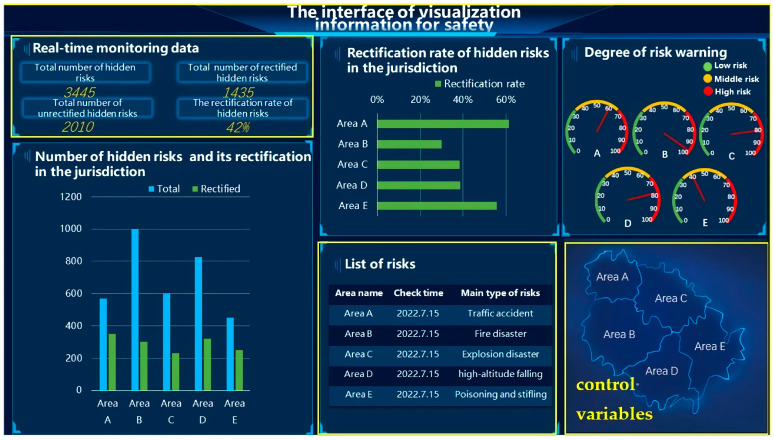
Control-variable modules in the UI design. Note. The yellow-line box shows the corresponding control modules.

To make the experimental situation more realistic, we added three control variables to the three-level UI design, as relevant auxiliary information (Figure 3). It was verified that the auxiliary control variables did not have a significant effect on the experimental data in the following analysis, and that subjects’ attention was significantly less allocated to these areas than to the main task areas.

The size and position of the VIS modules were controlled. Some studies have proposed that the layout of the visualization information in the interface influences the degree of information understandability and the accuracy and efficiency of people’s cognition process toward the human–machine interface, and that users are prone to pay more attention to the information on the left and the upper area [45]. To balance the impact of the layout of the main VIS-element presentation, we presented them at the bottom left, top center, and top right of the figure, and the size and position of the same modules were kept consistent across all UI levels.

The saturation of the red and green colors that represent “safe” and “danger” information in each UI level was controlled, and low saturation red and green was used to keep the colors of the stimulus consistent and avoid the strong visual impact of high saturation [17,46].

### 3.3. Experimental Design

Before the experiment, the specific content of the 3D-SART scale and the procedure of this experiment were briefly introduced to the subjects, who then entered the formal experiment. The procedures were as follows.

**Figure 4:**
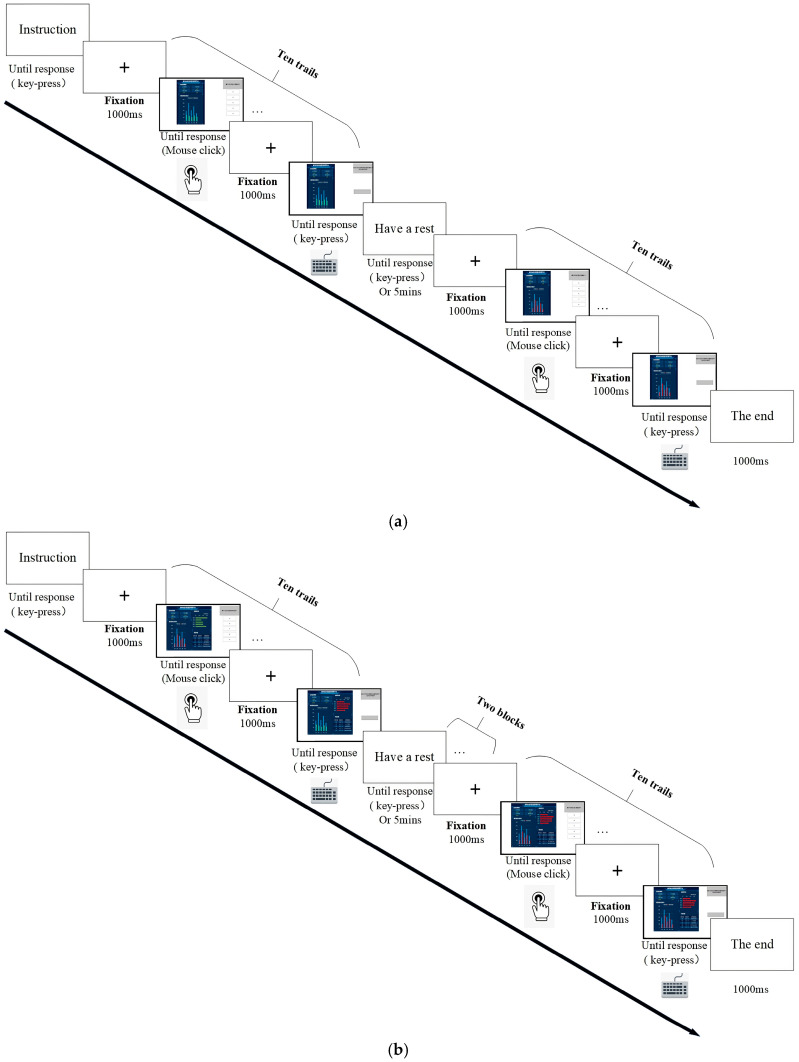

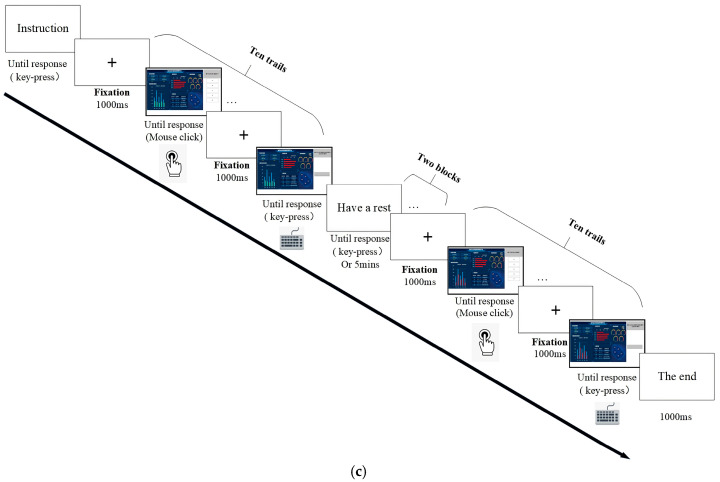
Experiment procedure: (**a**) The procedure of experiment UI1 (contains two contexts); (**b**) the procedure of experiment UI2 (contains four contexts); (**c**) the procedure of experiment UI3 (contains four contexts).

(1) The subject provided his/her informed consent.
(2) The subject sat in front of the screen with the eye tracker placed within an effective range of 45–75 cm, adjusted the seat and posture, and calibrated the gaze point, using the 5-point calibration method. When the subject entered the actual experiment, the eye-movement data were recorded simultaneously.
(3) The experiment involving a specific UI level (the between-subject variable) was randomly presented to the subject, who participated in only one level of interface, among which UI1 contained a total of two blocks, that is, two contextual frames, each block containing 10 trials. To distribute the learning effect, we randomly presented the contexts within the UI level, and the subject responded with a mouse click or key press, according to the corresponding probe questions. Each trial was preceded by a 1000-ms gaze point, “+”, to eliminate differences in the first-fixation position of subjects. After each situation, the subject would rest for 5 min and immediately complete the 3D-SART scale under the guidance of the experimenter, keeping the posture as still as possible during the process (Figure 4a). Both experiment UI2 and experiment UI3 consisted of four blocks, and the specific procedure was similar to that of UI1 (Figure 4b,c).
(4) At the end of the experiment, the subject was thanked and given a small gift.

### 3.4. Experimental Subjects

The sample size required for this study was calculated according to the G*power software, and the total sample size required to predict a level of statistical power of 80% at a significance level of α = 0.05 and a medium effect (f = 0.25) was obtained to be at least 159. We recruited 166 students from Xi’an University of Science and Technology and divided them into three groups to participate in this experiment. Based on Kihberger’s study of the framing effect, it was found that there was no significant difference between the findings obtained using the student population as a sample and those obtained using social workers with actual work experience [47]. Before the experiment, all participants were informed of the instructions and procedures, and provided their informed consent. All subjects had normal or corrected vision (no myopic astigmatism) and had sufficient sleep for the previous 24 h. The lighting conditions were kept as consistent as possible during all experiment sessions.

### 3.5. Laboratory Environment and Equipment

This study was approved by the Institution Review Board of the Research Center for Human Factors and Management Ergonomics, Xi’an University of Science and Technology, and it was performed following relevant ethical guidelines and regulations. The experimental task was designed using the E-prime 3.0 experimental platform, and the RED-m remote video-based eye tracker made by SensoMotoric Instruments in Teltow, Germany was used to collect ocular movements at a sampling rate of 60 Hz, with 0.5-degree calibration precision. The experimental materials were presented on a 15.6-inch laptop (Dell Precision M4800) with 1920 × 1080-pixel resolution, on which software was installed, including SMI iView RED-m and Begaze 3.7. The SMI iView RED-m was used to collect eye-movement data, which would be analyzed offline by Begaze 3.7.

### 3.6. Data Collection and Analysis

Statistical analyses were conducted on the behavioral-response data recorded by the E-prime 3.0 experimental platform, the SART scale data, and the eye-movement data recorded by SMI iView RED-m. The number of valid data points collected by the E-prime experimental platform for each UI level was 55, 55, and 56, and after excluding eight invalid eye-movement data points (i.e., data from eight participants with tracking ratios less than 90% and calibration accuracy higher than 1 degree were removed), we found that the number of valid eye-movement data points collected for each UI level was 51, 53, and 54. IBM SPSS Statistics 25.0 was used for statistical analysis, and the significance level used was α = 0.05. A multi-way ANOVA was used to determine the effect of the UI level and representation frame on the dependent variable. Post hoc tests were analyzed using Scheffé’s method, as the number of subjects varied between the UI levels [48,49].

## 4. Results

### 4.1. SPAM Measurement Results

The descriptive statistics and multi-way-ANOVA analyses were conducted using SPSS software on the three-stage and total scores of SA obtained by SPAM between UI levels of VIS and the presentation frames, and the results are shown in Table 3 and Table 4.

#### 4.1.1. Differences between UI Levels

**Figure 5:**
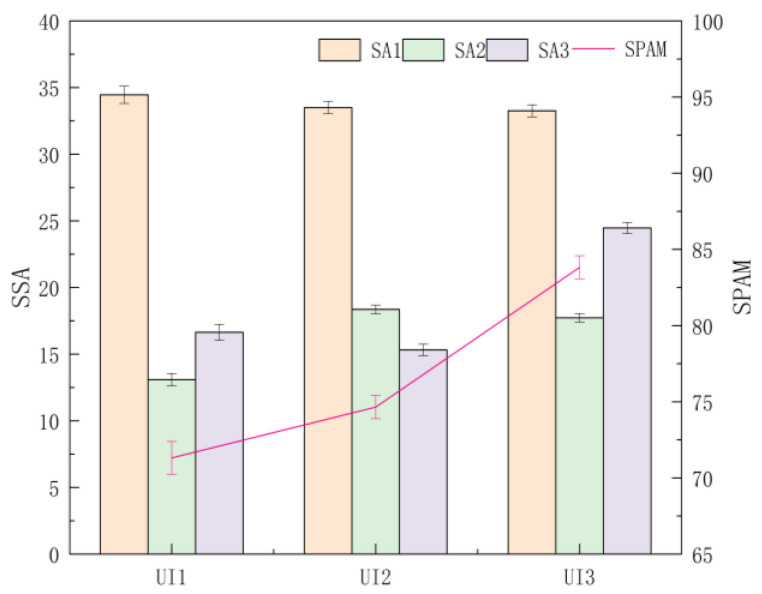
The stage and total scores of SA obtained by SPAM for each UI level. Note. SSA: the stage score of SA; SPAM: total score of SA; SA1: perception stage; SA2: comprehension stage; SA3: projection stage; Error bar: standard error.

A multi-way ANOVA was conducted on the three-stage SA theory, and the overall scores of SA between UI levels were obtained. It was found that the stage score of perception (SSA1) was not significantly different (F (2, 544) = 0.482, *p* = 0.618, $\eta_{p}^{2}$ = 0.002), the overall mean of which showed a trend of UI1 > UI2 > UI3. The stage score of comprehension (SSA2) was significantly different (F (2, 544) = 30.949, *p* < 0.001, $\eta_{p}^{2}$ = 0.102), and a post hoc test revealed that the SSA2 of UI2 and UI3 were significantly greater than that of UI1 (*p* < 0.001), with the overall mean showing a trend of UI2 > UI3 > UI1. The stage score of projection (SSA3) was significantly different (F (2, 544) = 126.546, *p* < 0.001, $\eta_{p}^{2}$ = 0.318), and a post hoc test revealed that the SSA3 of UI1 was significantly greater than that of UI2 (*p* = 0.012 < 0.05) and that the SSA3 of UI3 was significantly greater than that of UI1 and UI2 (*p* < 0.001), with the overall mean showing a trend of UI3 > UI1 > UI2. The total SPAM score was significantly different (F (2, 544) = 54.820, *p* < 0.001, $\eta_{p}^{2}$ = 0.168), and the post hoc test revealed that the total score of UI3 was significantly greater than that of UI1 and UI2 (*p* < 0.001), that the difference between UI1 and UI2 was significant (*p* = 0.042 < 0.05), and that the mean value of the total SPAM score was ranked UI3 > UI2 > UI1 (Figure 5).

#### 4.1.2. Differences between Presentation Frames

**Figure 6:**
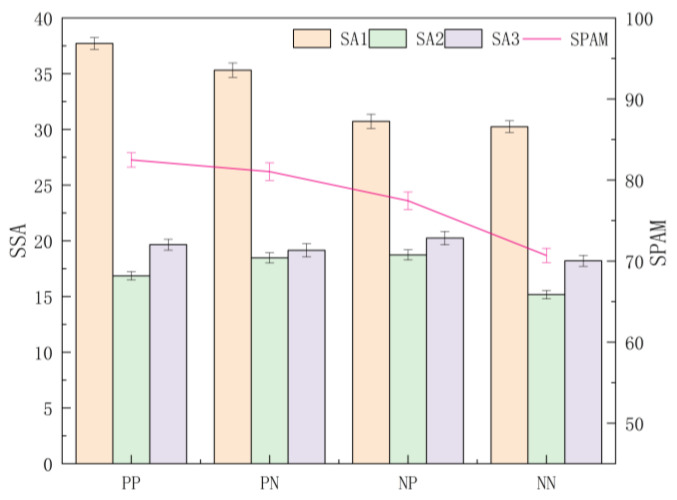
The stage and total scores of SA obtained by SPAM for each presentation frame. Note. PP: positive–positive frame; PN: positive–negative frame; NP: negative–positive frame; NN: negative–negative frame; SSA: the stage score of SA; SPAM: total score of SA; Error bar: standard error.

A multi-way ANOVA was conducted for three-stage and total SA scores under each presentation frame, and it was found that the differences in the perception stage score (SSA1) were significant (F (3, 544) = 42.580, *p* < 0.001, $\eta_{p}^{2}$ = 0.190), with post hoc tests finding significant differences between the frames except between the NP and NN frames, with the overall mean presented as PP > PN > NP > NN. The difference in the comprehension stage score (SSA2) was significant (F (3, 544) = 5.342, *p* = 0.001,$\;\eta_{p}^{2}$ = 0.029); post hoc tests found the SSA2 of NN frame significantly smaller than that of PP, PN, and NP frames (*p* < 0.001, *p* = 0.019 < 0.05); and the overall mean was NP > PN > PP > NN. By contrast, people scored higher SSA2 in the comprehension stage under a combination of positive and negative frames. For the projection stage score (SSA3), the differences were not significant across presentation frames (F (3, 544) = 2.132, *p* = 0.095, $\eta_{p}^{2}$ = 0.012), with the overall mean presented as NP > PP > PN > NN. For the total SPAM score, the differences were significant (F (3, 544) = 31.898, *p* < 0.001, $\eta_{p}^{2}$ = 0.150), and post hoc tests revealed that there were significant differences between the frames except for the PP and PN frames, NP frame, and PN frame, with the overall mean presented as PP > PN > NP > NN (Figure 6).

### 4.2. SART-Measurement Results

The descriptive statistic and multi-way-ANOVA analysis were conducted by SPSS software on the three-dimensional and total scores of SA obtained by SART between UI levels of VIS and the presentation frames, and the results are shown in Table 5 and Table 6.

#### 4.2.1. Differences between UI Levels

**Figure 7:**
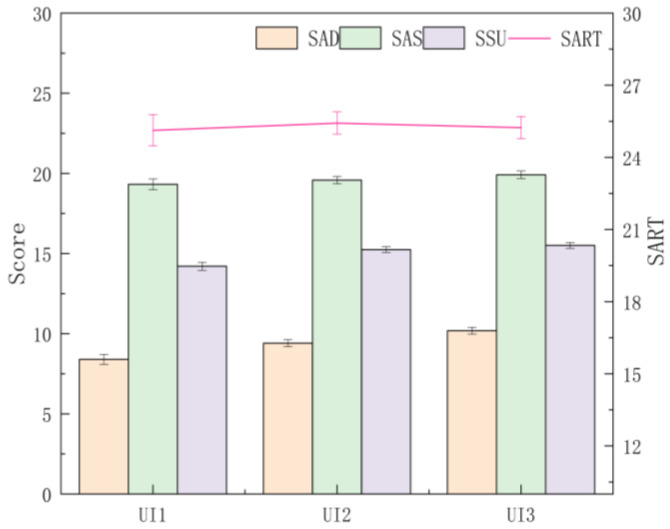
The dimension and total scores obtained by SART for each UI level. Note. Score: the dimension score of SA; SART: total score of SA; AD: attention demand dimension; AS: attention supply dimension; SU: situation understanding dimension; Error bar: standard error.

A multi-way ANOVA was conducted on each dimension, and total scores were obtained by SART among UI levels. The results show that the dimension scores of AD and SU (SAD and SSU) were significantly different among UI levels (F (2, 544) = 10.456, *p* < 0.001, $\eta_{p}^{2}$ = 0.037; F (2, 544) = 11.552, *p* < 0.001, $\eta_{p}^{2}$ = 0.041). Through a post hoc comparative analysis, it was found that UI3 was significantly greater than UI2 and UI1 in the SAD (*p* = 0.036 < 0.05, *p* < 0.001) and that there were significant differences between UI1 and UI2 (*p* = 0.027 < 0.05), with UI2 obtaining a higher score. It can be seen from the mean value that the SAD increased with the UI level, that is, UI3 > UI2 > UI1; for the SU dimension, the SSU of UI3 and UI2 were significantly greater than that of UI1 (*p* < 0.001, *p* = 0.003 < 0.05), and subjects’ acquisition and understanding of the information showed a trend of increasing with UI levels. For the dimension scores of AS (SAS), the differences were not significant between UI levels (F (2, 544) = 1.660, *p* = 0.191, $\eta_{p}^{2}$ = 0.006), and the mean value was UI2 > UI3 > UI1. There was no significant difference in the total score of SART (F (2, 544) = 0.361, *p* = 0.697, $\eta_{p}^{2}$ = 0.001), but the overall mean showed UI2 > UI3 > UI1 (Figure 7).

#### 4.2.2. Differences between Presentation Frames

**Figure 8:**
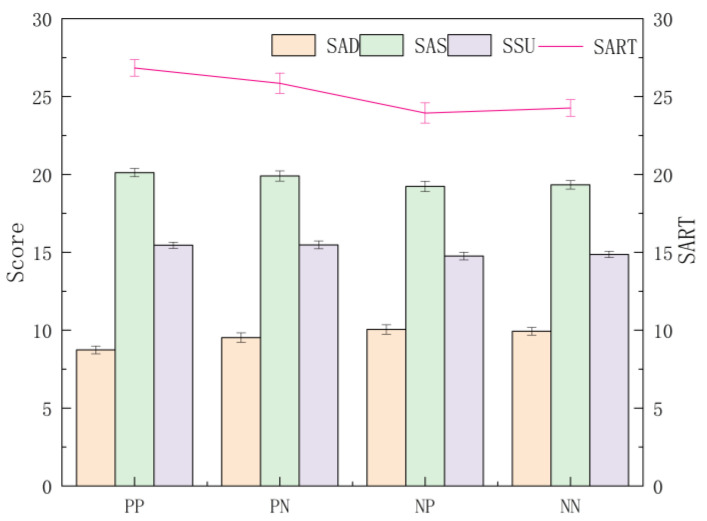
The dimension and total scores obtained by SART for each presentation frame. Note. Score: the dimension score of SA; SART: total score of SA; AD: attention-demand dimension; AS: attention-supply dimension; SU: situation-understanding dimension; PP: positive–positive frame; PN: positive–negative frame; NP: negative–positive frame; NN: negative–negative frame; Error bar: standard error.

A multi-way ANOVA was conducted on the dimension and total scores obtained by SART among the representation frames, and the results show significant differences in the AD dimension scores (F (3, 544) = 4.396, *p* = 0.005, $\eta_{p}^{2}$ = 0.024), with post hoc tests finding that the SAD of the PP frame was significantly smaller than NP and NN (*p* = 0.011 < 0.05, *p* = 0.009 < 0.05) and that the overall mean was presented as NP > NN > PN > PP; in the AS dimension, the differences were only marginally significant (F (3, 544) = 2.506, *p* = 0.058, $\eta_{p}^{2}$ = 0.014), and the mean values showed PP > PN > NN > NP. The differences between the SU dimension did not differ significantly across the presentation frames (F (3, 544) = 4.222, *p* = 0.006, $\eta_{p}^{2}$ = 0.023), but the mean values were presented as PN > PP > NP > NN for the SSU. Moreover, the total SART score differed significantly across the presentation frames (F (3, 544) = 5.922, *p* = 0.001 < 0.05, $\eta_{p}^{2}$ = 0.032), and post hoc tests found that PP was significantly greater than NP and NN (*p* = 0.008 < 0.05, *p* = 0.09 < 0.05); the overall mean value was presented as PP > PN > NN > NP (Figure 8). The subjective SA scores of subjects toward a certain situation differed under different combinations of the representation frames of VIS. When the frames were guided by the positive frame, the highest subjective SA score was obtained, and when the frames were guided by the negative frame, the lowest subjective score was obtained.

### 4.3. Overall-Risk Perception

To determine whether there are differences in subjects’ perceptions of risk under different representation frames, we conducted further statistical analysis on subjects’ scores on Q7 in each context (as shown in Table 7). A 7-point Likert scale was used to measure subjects’ overall-risk perception, with higher scores representing perceptions that the jurisdiction is safer and lower scores representing perceptions that the jurisdiction is more dangerous.

**Figure 9:**
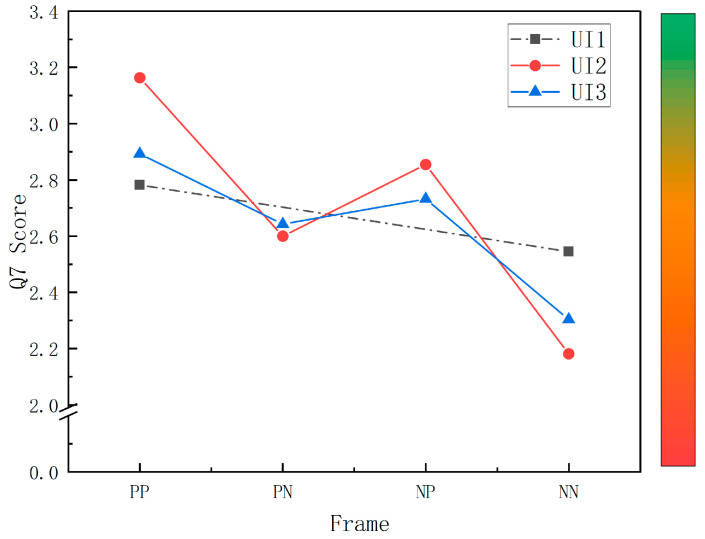
The interactive effects of Q7 score between UI-levels and presentation frames. Note. Non-estimable means are not plotted, so UI1 is represented by a dotted line: UI1 contains a small amount of VIS, and only two presentation frames exist in this interface, a positive frame (PP) and a negative frame (NN), with no combined frames (PN and NP); the colored bar represents the subject’s perceived level of risk (green = larger Q7 score and less perceived risk, red = smaller Q7 scores and more perceived risk).

A multi-way ANOVA on the Q7 scores revealed that the overall perception of risk did not differ significantly among UI levels (F (2, 544) = 0.297, *p* = 0.743, $\eta_{p}^{2}$ = 0.001), with the mean values showing a trend of UI2 > UI1 > UI3. However, the difference between presentation frames was significant (F (3, 544) = 15.201, *p* < 0.001, $\eta_{p}^{2}$ = 0.077), and a post hoc test revealed that the Q7 score of the PP frame was significantly greater than that of PN and NN (*p* = 0.021 < 0.05, *p* < 0.001), that NP was significantly greater than that of NN (*p* < 0.001), and that the mean score of Q7 was generally presented as PP > NP > PN > NN, with a higher Q7 score representing less perceived risk. The interactions between UI and frame were significant (F (4, 544) = 2.841, *p* = 0.024, $\eta_{p}^{2}$ = 0.020), and further simple-effects analysis revealed that the difference in perceived risk across presentation frames within UI1 was not significant (F (1, 544) = 2.166, *p* = 0.142 > 0.05, $\eta_{p}^{2}$ = 0.004), that the difference in perceived risk within UI2 was significant (F (3, 544) = 13.374, *p* < 0.001, $\eta_{p}^{2}$ = 0.069), and that the Q7 score of the PP frame was significantly greater than that of PN and NN (*p* = 0.003 < 0.05, *p* < 0.001). The Q7 score of the NP frame was significantly greater than that of the NN frame (*p* < 0.001); the difference in perceived risk across representation frames within UI3 was significant (F (3, 544) = 4.885, *p* = 0.002 < 0.05, $\eta_{p}^{2}$ = 0.026), and the Q7 score of the PP and NP frame was significantly greater than that of the NN frame (*p* < 0.001, *p* = 0.043 < 0.05) (Figure 9).

### 4.4. Analysis of Eye-Movement Indicators

#### 4.4.1. Overall Distribution of Attention

The valid eye-movement data of the subjects were imported into MATLAB R2022a, and a simple tool for examining fixations (ASTEF) developed by the laboratory of Nocera et al. was run [50]. Based on the spatial-statistical algorithm [51], the NNI was calculated to check the average dispersion of subjects’ gaze points in each context; the closer the mean value of NNI to 1, the more discrete the distribution of gaze points. A multi-way-ANOVA analysis was conducted on the NNI, and the results are shown in Table 8.

**Figure 10:**
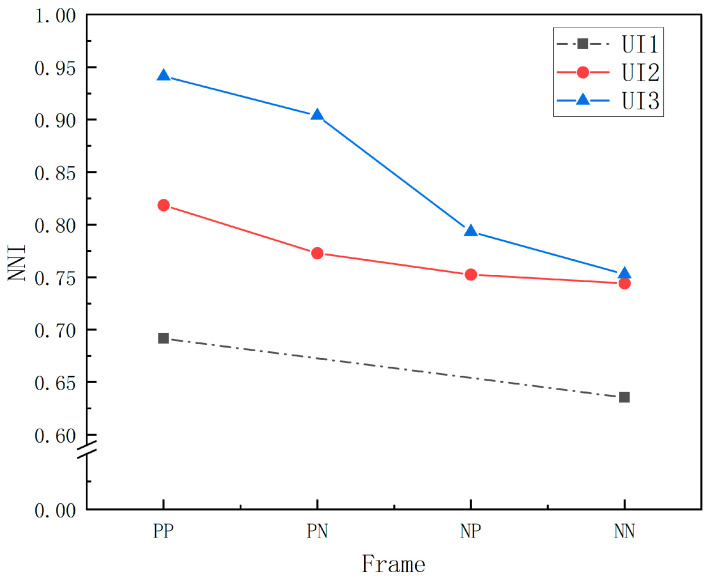
Interaction effects of NNI across UI levels and presentation frames. Note. Non-estimable means are not plotted, so UI1 is represented by a dotted line: UI1 contains a small amount of VIS, and only two presentation frames exist in this interface, a positive frame (PP) and a negative frame (NN), with no combined frames (PN and NP).

**Figure 11:**
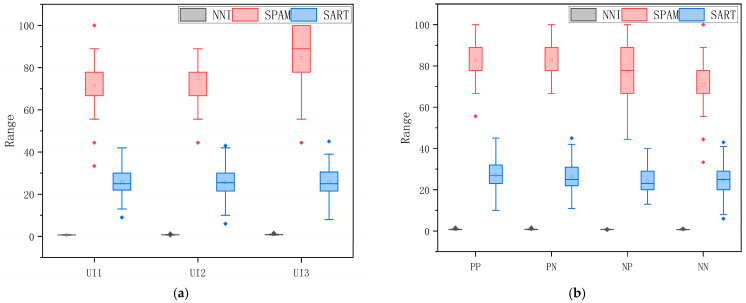
Correlation between NNI and SA scores obtained by SPAM and SART: (**a**) among UI levels; (**b**) among presentation frames.

The descriptive statistics showed that the mean values of subjects’ NNI fixation indexes were all less than 1, meaning that the subjects’ gaze points had an overall clustered distribution. A multi-factor ANOVA was conducted on the NNI for each UI level and presentation frame (Table 8), and it was found that the main effects were significant (F (2, 520) = 76.549, *p* < 0.001, $\eta_{p}^{2}$ = 0.227; F (3, 520) = 26.188, *p* < 0.001, $\eta_{p}^{2}$ = 0.131). Then, further post hoc tests and simple-effects analyses were carried out to obtain the following results: comparing the NNI of each UI level, the NNI of UI1 was significantly smaller than that of UI2 and UI3 (*p* < 0.001); the NNI of UI2 was significantly smaller than that of UI3 (*p* < 0.001), with the mean value specifically reflected as UI3 > UI2 > UI1. The NNI of the PP and PN frames was significantly larger than that of the NP and NN frames (*p* < 0.001), with the mean value specifically reflected as PN > PP > NP > NN. The interaction effect between each UI level and presentation frame was significant (F (4, 520) = 6.812, *p* < 0.001, $\eta_{p}^{2}$ = 0.050), as evidenced by the decreased NNI index from the positive guided frame to the negative frame within each UI level (Figure 10). The correlation between the NNI and the total SA score was further tested, and the results are shown in Table 9. It is shown that the Pearson correlation coefficient between NNI and SPAM score is r = 0.447 > 0.4 (*p* < 0.001), showing a significant moderate positive correlation; the Pearson correlation coefficient between NNI and SART score is r = 0.225 > 0.2 (*p* < 0.001), showing a significant low positive correlation (Figure 11).

#### 4.4.2. Partial Distribution of Attention

**Figure 12:**
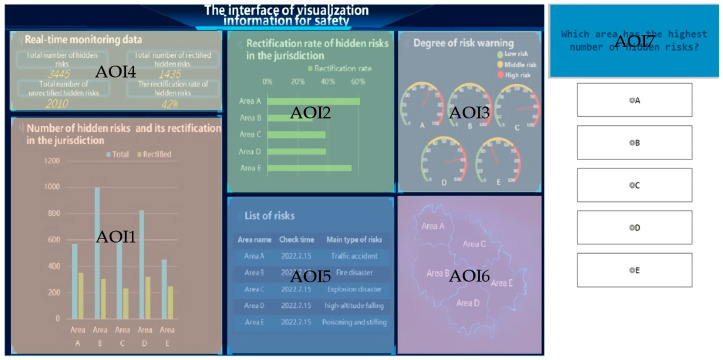
Interface AOI division.

There were seven divided areas of interest (AOIs), where AOI1 is the main perception module, AOI2 is the comprehension module, and AOI3 is the projection module; these are the main research modules of this paper. By contrast, AOI4, AOI5, and AOI6 are the control-variable modules of this paper, and AOI7 is the module for the presentation of SPAM probe questions, as shown in Figure 12.

**Figure 13:**
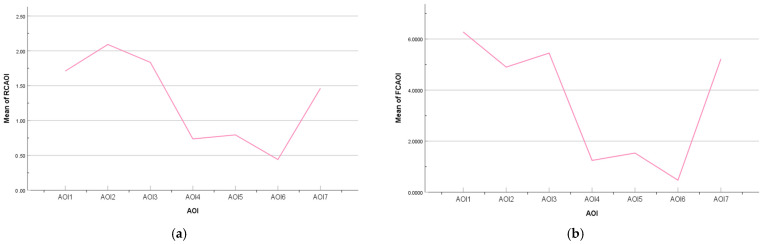
The partial distribution of attention among all the AOIs: (**a**) average regression count among AOIs; (**b**) average fixation count among AOIs.

A multi-way ANOVA was conducted on the average regression count (RCAOI), the percentage of fixation count (FCAOI), and the average fixation duration (FDAOI) in the AOI as dependent variables, and on the AOI and the three-stage score of SA as independent variables. A post hoc test revealed that for the RCAOI, FCAOI was significantly greater in AOI1, AOI2, AOI3, and AOI7 than in AOI4, AOI5, and AOI6, and to some extent, the use of AOI4, AOI5, and AOI6 as control variables in this paper was valid (as shown in Figure 13). Consequently, no statistical analysis was subsequently conducted for these three AOIs, and a multi-way ANOVA was only conducted for the indicators related to the key AOIs. The results of the descriptive statistics and multi-way analysis are shown in Table 10.

**Figure 14:**
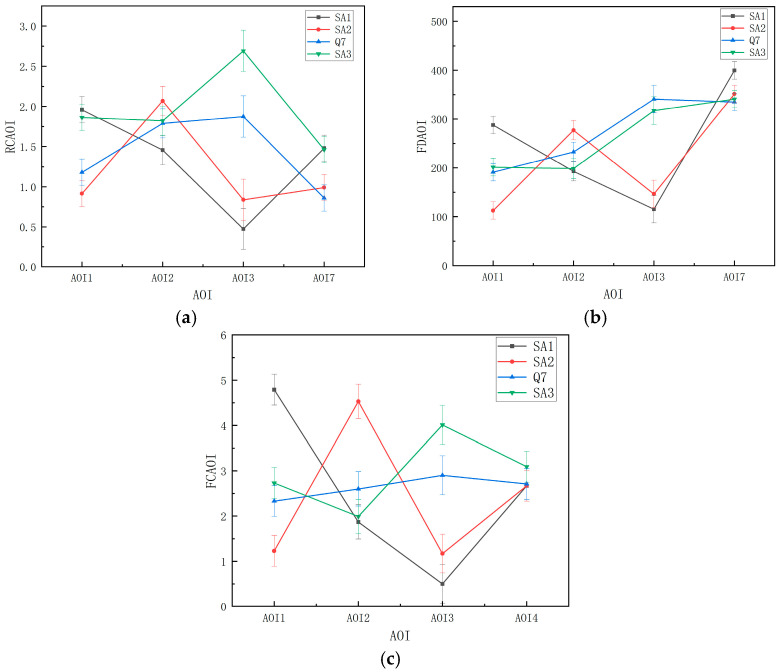
Interaction effect of attention allocation between the key AOIs and three stages of SA: (**a**) average regression count among key AOIs; (**b**) average fixation duration among key AOIs; and (**c**) average fixation count among key AOIs. Error bar: standard error.

From the results, it can be seen that for the RCAOI, there was a significant difference between the AOIs (F (3, 112) = 9.114, *p* < 0.001, $\eta_{p}^{2}$ = 0.196), with post hoc tests finding that the RCAOI of AOI2 was significantly greater than that of AOI1 and AOI7 (*p* = 0.024 < 0.05, *p* < 0.001), with the mean value presenting as AOI2 > AOI3 > AOI1 > AOI7. We also observed a significant difference between SA stages (F (3, 112) = 22.857, *p* < 0.001, $\eta_{p}^{2}$ = 0.380), with stage SA3 being significantly greater than stage SA2, with mean value of SA3 > Q7 > SA1 > SA2. For the FDAOI, the difference between the AOIs was significant (F (3, 112) = 34.109, *p* < 0.001, $\eta_{p}^{2}$ = 0.477), and the post-hoc-test analysis revealed that the gaze time in AOI7 was significantly greater than that of other AOIs (*p* < 0.001), that the overall mean value showed that AOI7 > AOI3 > AOI2 > AOI1, and that the difference between the SA stages was significant (F (3, 112) = 18.520, *p* < 0.001, $\eta_{p}^{2}$ = 0.332). Post-hoc-test analysis showed that the FDAOI of SA3 was significantly longer than that of SA2 (*p* < 0.001) and that the overall mean showed Q7 > SA3 > SA1 > SA2. For the FCAOI, it was not significant across AOIs (F (3, 112) = 1.797, *p* = 0.152, $\eta_{p}^{2}$ = 0.046), but it was significant among SA stages (F (3, 112) = 9.709, *p* < 0.001, $\eta_{p}^{2}$ = 0.206), with the overall mean showing SA3 > Q7 > SA1 > SA2. All the indicators mentioned above had a significant interaction effect (*p* < 0.001) between the two; that is, in the SA1, subjects paid more attention to AOI1; in the SA2, subjects paid more attention to AOI2; and in the SA3, subjects paid more attention to AOI3 (Figure 14).

## 5. Discussion

### 5.1. Discussion of SA Scores at Different UI Levels

The statistical analysis of the three stages of SA and total scores obtained by SPAM revealed that in SA1, with the improvement in the UI level, SSA1 decreased. The reason for this is that attention is the key link of this stage, and as the UI level increases, subjects need to perceive and process more information. Thus, they experience an overload of attention resources caused by the increased amount of information at the interface. As a result, a decrease in the level of perception [52], combined with the participants’ increased scores in the AD dimension, as measured by SART, could also be demonstrated. In the stage SA2, subjects scored the highest SSA2 on UI2, probably because UI2 provides the rectified rate information of hidden hazards to help subjects understand, based on UI1, and the amount of information displayed in UI2 is less than that of UI3, so the appropriate information does not easily confuse subjects’ judgment. Previous studies have found that excessive information display presents a greater cognitive load and interference to people regarding filtering, classifying, reorganizing, and integrating information, making people prone to perceptual and comprehension errors, which in turn leads to human-factor errors [53,54]. Therefore, if the threshold of information presentation can be found in the interface design, it can help people better understand the information, to some extent. In the SA3, the addition of the projection module to UI3 helped improve the subjects’ risk prediction. For the total SA score, it was shown that the higher the UI level, the better subjects’ SA. Scholar Zhi suggested that the decreasing effect of SA due to attention allocation can be substantially improved by enhancing the quality of the interface [55]. Although the increase in the amount of information displayed due to the increased UI level led to a decrease in subjects’ SSA1, as the logic and correlation between the information increased, the interface provided more objective and comprehensive information rather than less information, which contributed to subjects’ global perception, comprehension, and prediction, thus increasing subjects’ level of SA.

**Figure 15:**
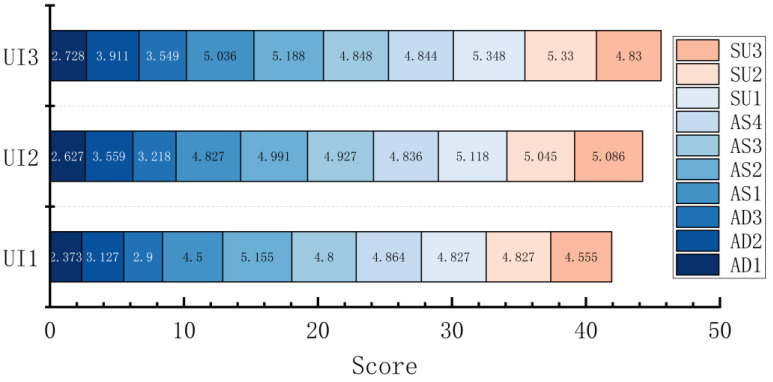
Ten sub-dimension scores of SART of each UI level.

The statistical analysis of the three-dimensional and total scores obtained by SART for each UI level showed that as the amount of VIS presented increased, the demand for subjects’ attention resources increased, with the SAD showing an increasing trend. However, to some certain extent, the VIS presentation interface, designed based on the three-stage SA theory, helped subjects better perceive, understand, and predict information, and thus the SSU showed an increasing trend with the increase in the UI level. In the AS dimension, the degree of mental arousal, concentration of attention, and total spare capacity of subjects in the corresponding situations showed a tendency for higher-level interfaces to achieve a higher score, while in the attention-allocation dimension, subjects subjectively perceived UI2 to be better (Figure 15). Relatively speaking, this may be because humans’ attention resources are limited [56], and with limited attentional resources, increased UI levels lead to an increase in the amount of information that subjects need to perceive and process, making it difficult for subjects to allocate their attention resources well. As the higher levels of UI provide a combination of information to aid comprehension, the overall supply of attention to the subject is increased. As for the overall SART scores, although there was no significant difference, the means indicated that subjects tended to feel that their perceptual comprehension and prediction ability were better and that their level of SA was higher, which was generally consistent with the SPAM results. Although some differences exist between UI2 and UI3, people subjectively may prefer the presentation of appropriate information, while the actual situation showed that the subjects had the best SA in UI3, which may be because of the amount of visual information displayed on the interface and because of the logic and relevance of the information.

### 5.2. Discussion of SA Scores for Different Presentation Frames

A statistical analysis of the three-dimensional and total scores obtained by SPAM showed that the total SA score, SSA1, and SSA2 decreased with the presentation frame of VIS from positive to negative. Combined with the Q7 scores, the level of overall perceived risk for the jurisdiction varied across the presentation frames. In the positive frame, subjects perceived less risk and had a higher level of SA, while in the negative frame, subjects perceived more risk and had a lower level of SA. The reason for the higher level of SA under the perception of less risk may be the fact that different presentation frames of VIS evoke different emotional attitudes of subjects, and thus lead to changes in their cognitive strategies and levels [57,58]. In a positive frame, people tend to perceive and judge positively and optimistically, with positive emotions facilitating the processing of information [59]. By contrast, a negative frame tends to be perceived in a negative and pessimistic manner, with negative emotions inhibiting cognitive processes by inhibiting automatic processing [60]. The framing effect was thus evident between positive and negative presentation frames. The reason for the higher SSA2 under the PN and NP frames compared with the PP and NN frames may be that subjects’ emotional attitudes were neutralized under the combined positive and negative presentation frames, thus enabling them to integrate relevant information more objectively. In SA3, the SSA3 was not significant across the presentation frames, further revealing that the effect of the framing effect was mainly concentrated in the perception and understanding stages of SA. Overall, the subjects’ risk-perception magnitude and level of SA showed a significant framing effect.

**Figure 16:**
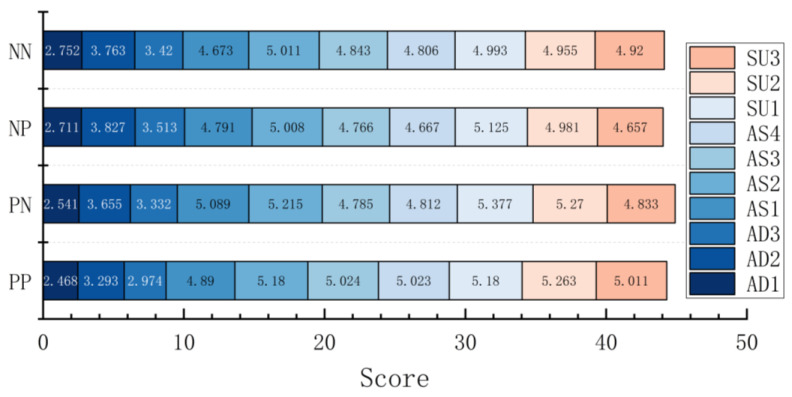
Ten sub-dimension scores of SART of each presentation frame.

A statistical analysis of the three-dimensional and total scores obtained by SART among representation frames showed that subjects’ subjective SAD was highest under the negative frame because people subjectively perceive this situation to be more complex, variable, and unstable, requiring more attention resources to make judgments and perceptions. Regarding the AS dimension, subjects’ degree of mental arousal, concentration of attention, and spare capacity in different presentation frames tended to be better when guided by the positive frame, while the distribution of attention tended to be worse in a positive and negative combination frame (Figure 16). This is probably because when information was presented in the positive–negative or negative–positive combination frames, it somewhat distracted subjects’ attention, and although subjects’ subjective intuitive level of AS was lower, subjects’ ability to comprehend information improved, owing to the comprehension and integration of the information presented, so the SPAM score reflected a trend of improvement. In sum, on the SU dimension and the overall SART score, there was a tendency for people to subjectively view the positive frame as superior to the negative frame, which is consistent with subjects’ performance of SA as reflected in the SPAM score.

### 5.3. Discussion of Attention Distribution

Based on the data of eye-movement indexes, the attention distribution of the subjects in each UI level and presentation frame was statistically analyzed. It was found that the overall spatial distribution of the subjects’ fixation points was aggregated. This is probably because the SPAM requires subjects to locate certain modules of VIS in the interface, to answer the corresponding probe questions. When individuals are driven by the search task, owing to limited cognitive resources, attention-selection mechanisms encourage individuals to focus on areas where they expect to receive relevant information, and create stimulus filters to prevent individuals from being distracted by irrelevant information [40], resulting in a more aggregate gaze. The NNI values of the subjects were larger at the higher UI levels and when the UI was guided by the positive frame, with a more discrete distribution of gaze points and a relatively higher level of SA. This may be because as the UI level increases, subjects need to allocate their limited attention to the additional VIS modules, thus distributing their attention more discretely, and the three-level UI design helps to increase their level of SA. The highest aggregation of gaze distribution in the negative frame may be due to negative information motivating decision makers to process information and to thus devote more attention to it, but it may also be due to people paying more attention to the red than the green stimuli. The reason the level of SA was higher when the distribution of gaze points was more discrete is that people tend to perceive and understand judgments from a global perspective, which is also in line with the research findings, to some extent [40,61,62].

Statistical analysis of the average number of regression counts, average fixation count, and average fixation duration of the AOIs showed that the subjects’ local-attention distribution in each situation was that they paid more attention to AOI1 in SA1, AOI2 in SA2, and AOI3 in SA3, and that there was no significant difference in their attention to the SPAM probe questions. It can be indicated that the subjects searched for and located the corresponding information in the interface for decision making based on the SPAM questions. However, it does not mean that the higher the level of decision making, the more likely it is that one can accurately locate the corresponding information module. The SART and SPAM scores showed that SA was influenced by different UI levels and presentation frames, and the phenomenon of subjects failing to make accurate decisions despite being able to accurately locate information proved the validity of this experiment, to a certain extent.

## 6. Limitations and Future Directions

The limitations and future research prospects for this study are as follows.

(1) This study investigated the framing effect of VIS. To make this study more practical, the safety and danger VIS elements were presented in green and red, respectively, based on the realistic situation and Chinese culture while manipulating the attributing frame of the VIS. Considering that presenting safety and danger information in the same color would have been out of context, different visual saliencies of the stimuli may also have had an impact on an individual’s visual processing, to a certain extent, which can be further investigated in the future by controlling the visual characteristics. Other features of VIS, such as color and shape, can also be further explored in the future, to investigate effective ways of VIS presentation and thus improve the stage level of SA.
(2) This study measured the cognitive processing of the subjects in different presentation frames using subjective scales (focusing on subjective behavioral performance) and eye-movement data (focusing on information-perception processes). In the future, the internal cognitive processing of individuals under different construction and presentation frames of VIS can be further explored in conjunction with EEG, in the three stages of information perceiving, understanding, and predicting, to further investigate the cognitive-processing differences of individuals.

## 7. Conclusions

This study took VIS as the research object, combined the theories related to SA and the framing effect, and designed a three-level VIS presentation interface. On this basis, we explored the framing effect of different VIS presentation frames, and at the same time, we used eye-movement indicators to measure the subjects’ attention allocation, which to some extent enriches the theoretical study of SA and the framing effect in the safety field. Specifically, the following conclusions were drawn from this study.

(1) For the construction of information, increasing the UI level effectively enhances the level of SA for the subjects. Although the increase in the amount of information displayed due to the increment in the UI level may lead to a decrease in subjects’ perceived level of SA and an increase in attentional demands, the higher-level interface helps to increase subjects’, as it fully considers the three stages of SA: perception, understanding, and prediction.
(2) There is a framing effect on the presentation of VIS, and the representation frame affects the individual’s SA. People perceive risk differently in different presentation frames, with people perceiving less risk in positive frames and more risk in negative frames. Subjects have better levels of perception and prediction of information, and the highest levels of SA, in the fully positive frame, better levels of comprehension in the combined frame, and the lowest levels of SA in the fully negative frame.
(3) To a certain extent, the NNI provides a process index for this study, quantifying the subjects’ eye-movement gaze strategies. The more discrete the distribution, the better the subjects can integrate information from a global perspective, and the higher the level of SA. The validity of this experiment is further supported by other eye-movement metrics from the perspective of the partial distribution of attention.

This study can provide some reference for the design and optimization of the VIS presentation interface. When designing the VIS presentation interface, researchers should ensure that the amount of information displayed on the interface is moderate; at the same time, the logic and correlation between the information on the interface should be improved; that is, the layout of the VIS presentation interface should fully consider the three stages of SA, so that the interface level can conform to the three stages of cognitive processing of people’s information and help people improve their SA. For the selection of the presentation frame of VIS, the positive and negative frames should be combined as much as possible, so that people can better integrate relevant information, make judgments on the current safety-risk situation, and improve SA.

## Figures and Tables

**Table 1:** SPAM question library.

Stage of SA	NO.	Questions	Answer
SA1	1	Which area has the highest number of hidden risks?	B
	2	Which area has the lowest number of hidden risks?	E
	3	Which area has the lowest number of hidden risks rectified?	C
	4	Which area has the highest number of hidden risks rectified?	A
SA2	5	Which area has the lowest rectification rate of hidden risks?	B
	6	Which area has the highest rectification rate of hidden risks?	A
	7	What is the overall risk profile of the whole jurisdiction?	1 (very dangerous)–7 (very safe)
SA3	8	Which area is most likely to see a safety-risk accident?	B
	9	Which area is least likely to see a safety-risk accident?	E
	10	What is the ranking of the likelihood of a safety accident in the jurisdiction? (Ranked from most to least prone to see a safety-risk accident)	BCDAE

**Table 2:** SART scale.

Dimension	NO.	Sub-Dimension	Items
AD	1	Degree of situation instability	How stable is the context?
	2	The complexity of the situation	What is the complexity of the situation?
	3	The degree of variability in the situation	How many variables are present in the situation?
AS	4	Level of mental arousal	How aroused are you in this situation?
	5	Level of attention concentration	To what extent are you able to focus your attention on the situation?
	6	Level of attention division	Are you able to pay attention to a lot of information at one time?
	7	Level of spare capacity	How much spare capacity do you have left in the situation?
SU	8	Quantity of information obtained	How much of the information in the situation are you able to access and understand?
	9	Quality of information obtained	What is the quality of the information you receive and understand?
	10	Familiarity with the context	How familiar are you with the situation?

Note. AD: attention demand; AS: attention supply; SU: situation understanding.

**Table 3:** The stage and total-SA scores obtained by SPAM.

UI Level	Presentation Frame	SSA1	SSA2	SSA3	SPAM	Number of Subjects
		Mean ± SD	Mean ± SD	Mean ± SD	Mean ± SD	
UI1	PP	38.36 ± 4.200	13.82 ± 5.608	18.00 ± 5.578	77.98 ± 9.440	55
	NN	30.55 ± 7.798	12.36 ± 6.929	15.27 ± 7.163	64.65 ± 12.667	55
	Total	34.45 ± 7.368	13.09 ± 6.317	16.64 ± 6.535	71.31 ± 12.980	110
UI2	PP	37.45 ± 5.170	18.55 ± 3.558	14.91 ± 5.733	78.79 ± 7.790	55
	PN	35.64 ± 5.362	18.91 ± 3.146	14.73 ± 5.039	76.97 ± 8.241	55
	NP	30.91 ± 8.665	18.55 ± 4.045	17.27 ± 4.889	74.14 ± 12.478	55
	NN	30.00 ± 7.454	17.45 ± 5.517	14.36 ± 6.013	68.69 ± 11.334	55
	Total	33.50 ± 7.465	18.36 ± 4.171	15.32 ± 5.522	74.65 ± 10.790	220
UI3	PP	37.32 ± 5.219	18.21 ± 3.865	26.07 ± 5.284	90.68 ± 9.422	56
	PN	35.00 ± 7.628	18.04 ± 4.439	23.57 ± 6.723	85.12 ± 11.849	56
	NP	30.54 ± 6.444	18.93 ± 3.121	23.21 ± 8.551	80.76 ± 13.314	56
	NN	30.18 ± 8.200	15.71 ± 6.566	25.00 ± 7.385	78.77 ± 14.413	56
	Total	33.26 ± 7.553	17.72 ± 4.800	24.46 ± 7.130	83.83 ± 13.132	224
Total	PP	37.71 ± 4.880	16.87 ± 4.906	19.70 ± 7.255	82.53 ± 10.616	166
	PN	35.32 ± 6.581	18.47 ± 3.861	19.19 ± 7.402	81.08 ± 10.968	111
	NP	30.72 ± 7.593	18.74 ± 3.597	20.27 ± 7.563	77.48 ± 13.270	111
	NN	30.24 ± 7.781	15.18 ± 6.672	18.25 ± 8.382	70.75 ± 14.123	166

Note. SSA1: the stage score of SA1; SSA2: the stage score of SA2; SSA3: the stage score of SA3; SPAM: total score of SA; PP: positive–positive frame; PN: positive–negative frame; NP: negative–positive frame; NN negative–negative frame; SD: standard deviation.

**Table 4:** Multi-way-ANOVA-analysis results of each stage and the total score of SPAM.

Source	Dependent Variable	Type III Sum of Squares	df	Mean Square	F	Sig.	Partial Eta Squared
UI	SSA1	44.263	2	22.131	0.482	0.618	0.002
	SSA2	1461.981	2	730.991	30.949	<0.001	0.102
	SSA3	10,180.866	2	5090.433	126.546	<0.001	0.318
	SPAM	14,016.264	2	7008.132	54.820	<0.001	0.168
Frame	SSA1	5862.853	3	1954.284	42.580	<0.001	0.190
	SSA2	378.501	3	126.167	5.342	0.001	0.029
	SSA3	257.312	3	85.771	2.132	0.095	0.012
	SPAM	12,233.303	3	4077.768	31.898	<0.001	0.150
UI × Frame	SSA1	15.020	4	3.755	0.082	0.988	0.001
	SSA2	69.011	4	17.253	0.730	0.571	0.005
	SSA3	529.437	4	132.359	3.290	0.011	0.024
	SPAM	537.428	4	134.357	1.051	0.380	0.008

Note: SSA1: the stage score of SA1; SSA2: the stage score of SA2; SSA3: the stage score of SA3; SPAM: total score of SA.

**Table 5:** The dimension and the total scores obtained by SART.

UI Level	Presentation Frame	SAD	SAS	SSU	SART	Number of Subjects
		Mean ± SD	Mean ± SD	Mean ± SD	Mean ± SD	
UI1	PP	7.62 ± 3.088	19.73 ± 3.274	14.53 ± 2.899	26.64 ± 6.873	55
	NN	9.18 ± 3.074	18.91 ± 3.540	13.89 ± 3.077	23.62 ± 7.230	55
	Total	8.40 ± 3.166	19.32 ± 3.419	14.21 ± 2.993	25.13 ± 7.183	110
UI2	PP	8.91 ± 3.032	20.18 ± 3.394	15.80 ± 2.288	27.07 ± 6.675	55
	PN	9.15 ± 2.752	19.95 ± 3.223	15.69 ± 2.559	26.49 ± 6.330	55
	NP	9.67 ± 3.163	19.05 ± 3.498	14.51 ± 2.538	23.89 ± 6.554	55
	NN	9.89 ± 3.298	19.15 ± 4.093	15.00 ± 2.912	24.25 ± 8.168	55
	Total	9.40 ± 3.072	19.58 ± 3.577	15.25 ± 2.620	25.43 ± 7.057	220
UI3	PP	9.68 ± 3.197	20.45 ± 3.291	16.04 ± 2.508	26.80 ± 6.274	56
	PN	9.91 ± 3.492	19.86 ± 3.170	15.27 ± 2.408	25.21 ± 6.851	56
	NP	10.43 ± 3.230	19.41 ± 3.085	15.02 ± 2.378	24.00 ± 6.090	56
	NN	10.73 ± 3.539	19.95 ± 3.993	15.71 ± 2.455	24.93 ± 7.221	56
	Total	10.19 ± 3.371	19.92 ± 3.401	15.51 ± 2.453	25.24 ± 6.657	224
Total	PP	8.74 ± 3.204	20.12 ± 3.314	15.46 ± 2.646	26.84 ± 6.572	166
	PN	9.53 ± 3.156	19.90 ± 3.182	15.48 ± 2.482	25.85 ± 6.599	111
	NP	10.05 ± 3.205	19.23 ± 3.286	14.77 ± 2.460	23.95 ± 6.296	111
	NN	9.94 ± 3.352	19.34 ± 3.886	14.87 ± 2.907	24.27 ± 7.524	166

Note. SAD: the dimension score of attention demand; SAS: the dimension score of attention supply; SSU: the dimension score of situational understanding; SART: total score of SA; PP: positive–positive frame; PN: positive–negative frame; NP: negative–positive frame; NN: negative–negative frame; SD: standard deviation.

**Table 6:** Muti-way-ANOVA-analysis results of each dimension and the total score of SART.

Source	Dependent Variable	Type III Sum of Squares	df	Mean Square	F	Sig.	Partial Eta Squared
UI	SAD	213.485	2	106.742	10.456	<0.001	0.037
	SAS	40.005	2	20.003	1.660	0.191	0.006
	SSU	157.761	2	78.880	11.552	<0.001	0.041
	SART	33.855	2	16.927	0.361	0.697	0.001
Frame	SAD	134.621	3	44.874	4.396	0.005	0.024
	SAS	90.567	3	30.189	2.506	0.058	0.014
	SSU	86.496	3	28.832	4.222	0.006	0.023
	SART	833.414	3	277.805	5.922	0.001	0.032
UI × Frame	SAD	5.603	4	1.401	0.137	0.969	0.001
	SAS	11.197	4	2.799	0.232	0.920	0.002
	SSU	20.517	4	5.129	0.751	0.558	0.005
	SART	64.533	4	16.133	0.344	0.848	0.003

Note. SAD: the dimension score of attention demand; SAS: the dimension score of attention supply; SSU: the dimension score of situational understanding; SART: total score of SA.

**Table 7:** Tests of between-subjects effects. Dependent Variable: Q7.

Source	Type III Sum of Squares	df	Mean Square	F	Sig.	Partial Eta Squared
UI	0.422	2	0.211	0.297	0.743	0.001
Frame	32.340	3	10.780	15.201	<0.001	0.077
UI × Frame	8.061	4	2.015	2.841	0.024	0.020

**Table 8:** Tests of between-subjects effects. Dependent Variable: NNI.

Source	Type III Sum of Squares	df	Mean Square	F	Sig.	Partial Eta Squared
UI	2.215	2	1.107	76.549	<0.001	0.227
Frame	1.136	3	0.379	26.188	<0.001	0.131
UI × Frame	0.394	4	0.099	6.812	<0.001	0.050

**Table 9:** NNI and SPAM correlation table.

		NNI	SPAM	SART
NNI	Pearson Correlation	1	0.447 **	0.270 **
	Sig. (2-tailed)		<0.001	< 0.001
	N	530	530	530
SPAM	Pearson Correlation	0.447 **	1	0.225 **
	Sig. (2-tailed)	<0.001		< 0.001
	N	530	530	530
SART	Pearson Correlation	0.270 **	0.225 **	1
	Sig. (2-tailed)	<0.001	<0.001	
	N	530	530	530

** Correlation is significant at the 0.01 level (two-tailed).

**Table 10:** Results of the multivariate-ANOVA-main-effects test for each AOI eye-movement indicator.

Independent Variable	Dependent Variable	Type III Sum of Squares	df	Mean Square	F	Sig.	Partial Eta Squared
AOI	RCAOI	7.223	3	2.408	9.114	<0.001	0.196
	FDAOI	263,442.561	3	87,814.187	34.109	<0.001	0.477
	FCAOI	38.821	3	12.940	1.797	0.152	0.046
SA	RCAOI	18.116	3	6.039	22.857	<0.001	0.380
	FDAOI	143,035.259	3	47,678.420	18.520	<0.001	0.332
	FCAOI	209.763	3	69.921	9.709	<0.001	0.206
AOI × SA	RCAOI	9.531	9	1.059	4.008	<0.001	0.244
	FDAOI	133,411.300	9	14,823.478	5.758	<0.001	0.316
	FCAOI	295.330	9	32.814	4.556	<0.001	0.268

Note. RCAOI: average number of regression counts in area of interest; FDAOI: average fixation duration in area of interest; FCAOI: average number of fixation counts in area of interest.

## Data Availability

All the data in this study are presented.
